# A Continuous Manufacturing Approach for Aligned PVDF Nanofiber Yarns with Enhanced Mechanical and Piezoelectric Properties

**DOI:** 10.1021/acsapm.5c00069

**Published:** 2025-04-25

**Authors:** Adaugo Enuka, Mohamad Keblawi, Emmet Sedar, Vince Beachley

**Affiliations:** 1 Department of Chemical Engineering, Rowan University, Glassboro, New Jersey 08028, United States; 2 Department of Biomedical Engineering, Rowan University, Glassboro, New Jersey 08028, United States; 3 Department of Mechanical Engineering, Rowan University, Glassboro, New Jersey 08028, United States

**Keywords:** piezoelectric materials, electrospun PVDF-HFP nanofibers, postdrawing, nanofiber yarns, smart textiles

## Abstract

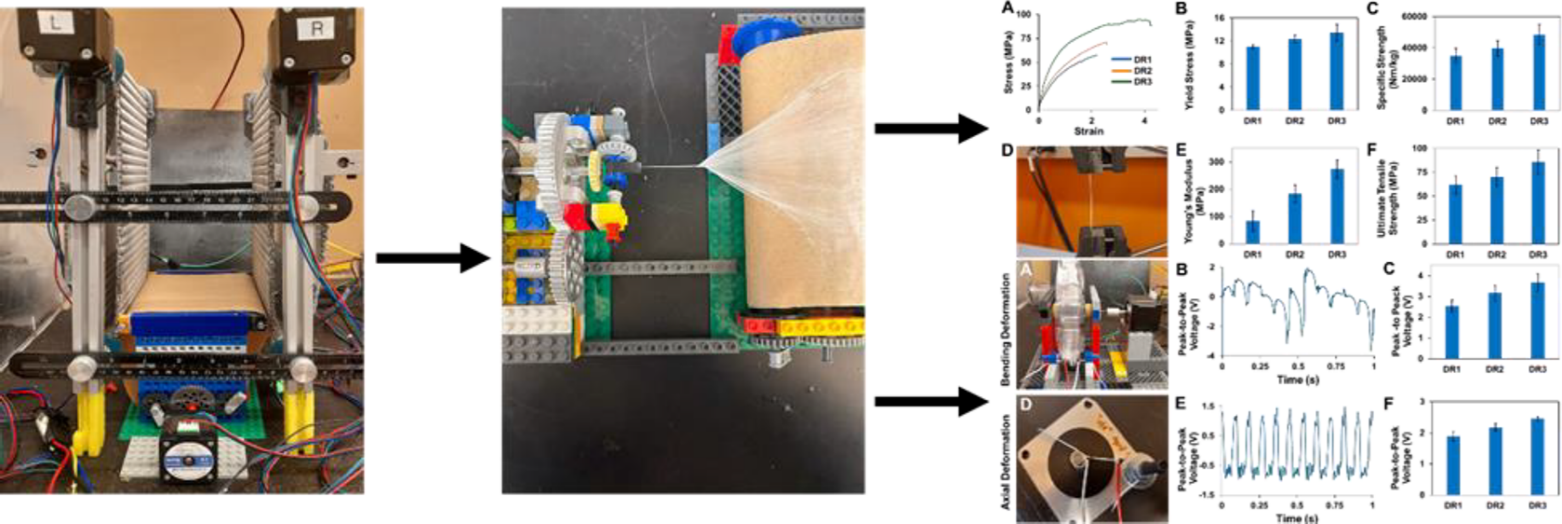

Electrospun poly(vinylidene fluoride-*co*-hexafluoropropylene) (PVDF-HFP) nanofibers possess desirable mechanical and piezoelectric properties, making them promising candidates for smart textiles if they can be assembled into continuous yarns. This study presents a manufacturing approach that enables the production of electrospun PVDF-HFP nanofiber yarns using an automated parallel track system and an adjustable roll-to-roll collector. Results show that this approach has potential for PVDF yarn manufacturing on a commercial scale. Electrospun yarns have previously been fabricated with self-bundling methods, but current technologies are limited by production limitations such as the lack of tight control over assembly parameters and the absence of a postdrawing process. Postdrawing was applied here to individual fibers before yarn spinning to enhance fiber strength by over two times and yarn strength by 39%. The piezoelectrical performance of yarns was enhanced by up to 45% with postdrawing. Continuous PVDF-HFP yarns with specific strength approaching 50,000 N m/kg and a relative β phase content of 97% are promising candidates for piezoelectric nanofiber-based smart textiles, which can be integrated into various wearable devices and intelligent garments.

## 1 Introduction

Electrospinning, as a versatile technology, provides a new avenue for developing smart polymer fiber materials.^1^ Smart textiles are advanced materials that can detect and react to their surroundings reliably and beneficially.^2^ Electrospinning has garnered significant attention over the past few decades due to its simple method of producing micro- and nanoscale fibers.^3,4^ Stimuli-responsive polymers engineered into nanoscale fibers via electrospinning can result in a larger surface area/volume ratio, leading to more interactive sites and enhanced sensitivity. Smart properties can also come from special polymers with piezoelectric and triboelectric effects, making them an excellent choice for flexible electronics and smart textile fabrication.^1^

Polyvinylidene fluoride (PVDF) is a well-known synthetic polymer with exceptional piezoelectric properties, first discovered in 1969. There are five known crystalline phases of PVDF: α, β, γ, δ, and ε. These phases determine the electroactive properties, with the β-phase being the most crucial due to its excellent piezo-, pyro-, and ferroelectric characteristics. In the β-phase, all dipole moments are aligned in the same direction, resulting in a nonzero dipole moment and the highest piezoelectric response.^5−7^ The β-phase can be induced in PVDF through a poling process commonly performed by subjecting a PVDF film to a high-voltage electrical field at elevated temperature.^8^ Since electrospinning inherently induces poling due to stretching via a strong electric field, no separate process is necessary to induce the β-phase.^9,10^ In addition to poling, PVDF has been combined with other materials to enhance their piezoelectric performance and create composite materials.^11^

Nonwoven nanofibers produced by electrospinning have poor mechanical strength, which limits their application in the field of textiles.^12−14^ Woven and knitted textiles represent ideal alternatives; however, both rely on high-quality continuous nanofiber yarns that are challenging to produce. Research efforts to directly produce nanofiber yarns from electrospinning have been going on for years.^15,16^ A widely used electrospun nanoyarn production technique is self-bundling electrospinning, where two oppositely charged polymer solution jets self-bundle into a nanoyarn. To achieve fiber alignment within the yarn, a grounded rotating funnel is added to an electrospinning setup. This approach is referred to as “cone spinning”.^14,17^ Another approach deposits electrospun nanofibers into a vortexed water path in order to twist them into nanoyarns.^18^ Despite these advancements, current techniques have yet to yield nanofiber yarns with the requisite length, mechanical properties, and uniformity that are essential for integration into commercial textile manufacturing processes.

One reason for the poor mechanical properties of nanofiber yarns is the lack of a postdrawing process during nanofiber fabrication. Conventionally spun polymer microfibers are significantly stronger than electrospun nanofibers, and it is hypothesized that these differences are a result of the postdrawing process present in the manufacture of conventional fibers.^19^ The principle of postdrawing involves applying a tensile force that elongates fibers after they are initially spun. The postdrawing step induces molecular orientation and crystalline changes that significantly improve the mechanical properties of polymer fibers.^20^ Postdrawing is naturally applied by an electrospinning jet when they are initially formed at high strain rates under the influence of an electrostatic force. However, during the later stages of electrospinning and after nanofiber deposition, the polymer chains within the nanofibers may rapidly revert to disordered orientation due to high polymer chain relaxation rates mediated by the solvent remaining in the fibers after strain is no longer occurring.^19^

Postdrawing of electrospun nanofibers is crucial as it enhances their mechanical properties to match or surpass conventionally spun fibers, combining high performance with the unique advantages of nanoscale structures such as high surface area.^20−22^ A parallel track collecting system enables electrospun nanofiber postdrawing to enhance strength by up to seven times.^21^ Postdrawing also enhances piezoelectric performance of electrospun nanofibers.^20^ Combining a parallel track electrospinning system with a roll-to-roll collector facilitates the assembly of postdrawn nanofibers into continuous yarns.^23^

This study investigates a PVDF copolymer poly(vinylidene fluoride-*co*-hexafluoropropylene) (PVDF-HFP) that exhibits enhanced solubility, hydrophobicity, and mechanical strength due to the incorporation of HFP in its molecular structure.^24−27^ The research question is based on manufacturing and investigates the ability to produce piezoelectric yarns and the effect of the fibers’ post draw ratio on the nanofiber yarn’s mechanical strength, crystal phases, and piezoelectric response.

Ultimately, this research endeavors to pave a way for manufacturing high-performance electrospun PVDF-HFP nanofiber yarns at a scale, unlocking their potential for different commercial applications and advancing piezoelectric nanofiber technology.

## 2 Materials and Methods

### 2.1 Preparation of PVDF-HFP Solution

PVDF-HFP copolymer pellets with a molecular weight of 400,000 Da (Sigma-Aldrich-427160) and organic solvents *N*,*N*-dimethylformamide (DMF, 99.8%, Beantown Chemical-136300) and acetone (99.5%, VWR-BDH1101) were used to make the PVDF-HFP electrospinning solution. A 30% w/v PVDF-HFP solution was prepared by mixing 5.4 g of PVDF-HFP pellets in a 2:1 DMF:acetone solvent system. This mixture was placed on a heated shaker at 65 °C for 24 h to ensure complete dissolution of the pellets. After 24 h, a viscous, homogeneous solution was obtained. Proper dissolution of the PVDF-HFP copolymer in the solvent mixture is crucial for obtaining a uniform solution suitable for electrospinning of nanofibers. Control polycaprolactone (PCL) solutions contained 18% PCL (Sigma-Aldrich-440744) w/v dissolved in 3:1 dichloromethane (99.7%, Beantown Chemical-145765):DMF.

### 2.2 Electrospinning

A high-voltage power supply (ES40P-10W Gamma High Voltage Research Inc.) applied 9 kV to a 21-gauge blunt capillary needle at a distance of 10 cm above the top of an automated track electrospinning collection system.^21^ A syringe pump (New Era Pump Systems) expelled the solution at a constant rate of 1 mL/h. The electrospinning process was performed in a humidity-controlled acrylic environmental chamber. Samples were collected within an observed temperature and humidity range of 20–25 °C and 50–60%, respectively.

### 2.3 Automated Parallel Track System and Postdrawing

**Figure 1:**
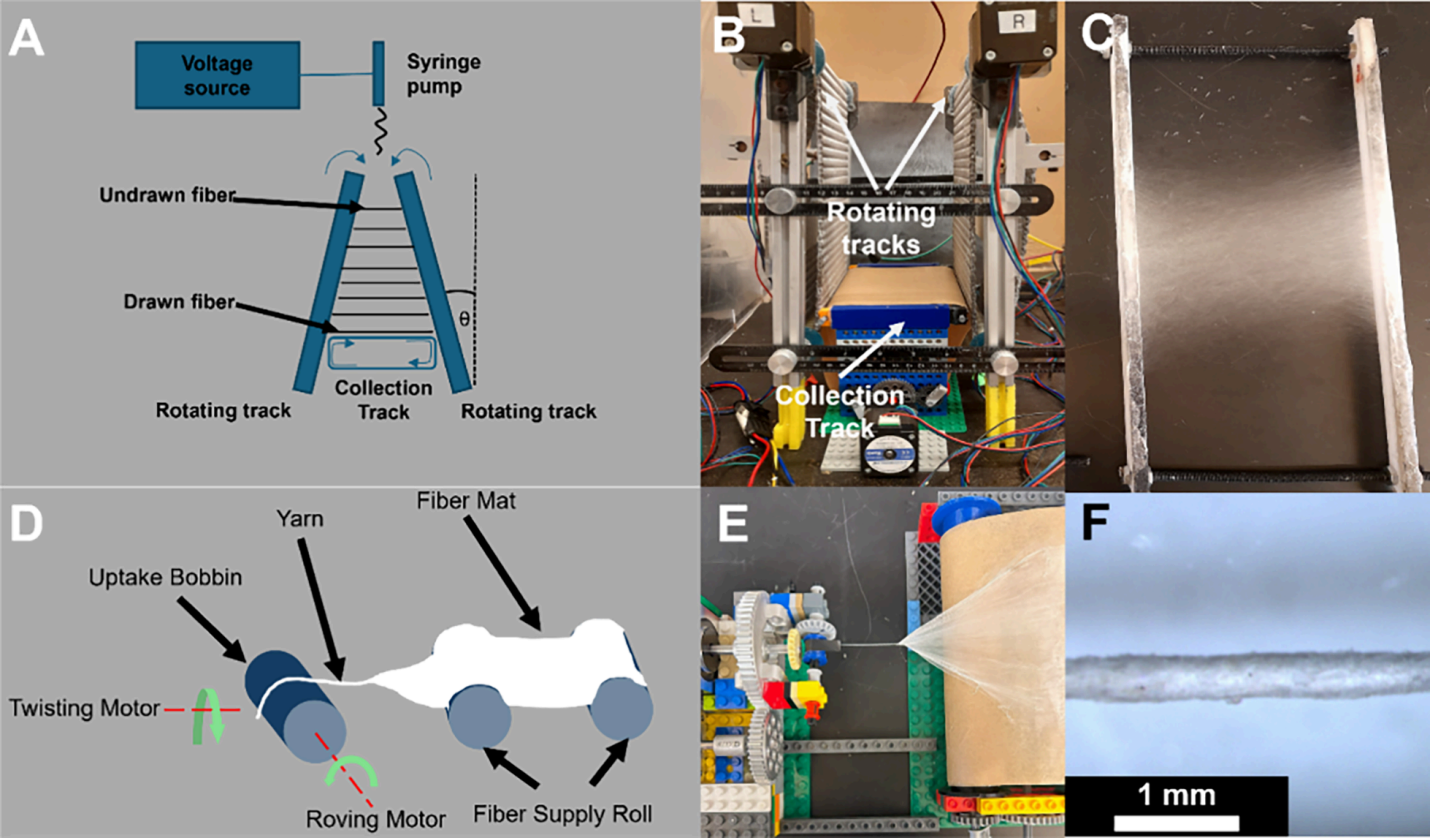
(A) Schematic and (B) photo of the electrospinning automated track system for facilitating the continuous collection and postdrawing of aligned electrospun nanofibers. The track angle (θ) can be adjusted to change the fiber DR. The top gap represents the undrawn nanofibers, while the bottom gap shows the postdrawn nanofibers. In (B), θ = 0° so the fibers are not drawn (DR1). Aligned nanofibers travel down the track until they are detached from the tracks and transferred to a roll-to-roll collection track. (C) A static tray collector is used (instead of the collection track) to assemble nanofiber control samples. (D) Schematic and (E) image of a continuous yarn being spun from a roll of aligned nanofibers. (F) Photo of a PVDF-HFP nanofiber yarn.

Postdrawing of electrospun nanofibers was done using an automated track collection system^21^ (Figure 1A,B). The angle of the tracks (θ) can be adjusted to apply a controlled axial strain that elongates the fibers immediately after electrospinning. This allows the postdrawing process to be integrated with electrospinning in a continuous manufacturing approach.

The electrospun aligned nanofibers suspend with one end adhered to each of the parallel tracks and then travel downward with track motion. Fibers shear off the tracks and deposit on a roll-to-roll collection track for yarn manufacturing (Figure 1A,B). Aligned nanofiber control samples (not spun into yarns) were collected by using a static rack (Figure 1C) in place of the collection track. The track elements of the assembly system were set at different angles (θ) to vary the gap length between the tracks at the top and bottom of the device. The top and bottom gaps between the tracks were 12 cm–12 cm, 6 cm–12 cm, and 4 cm–12 cm, respectively, to post draw the fibers at a draw ratio of 1:1, 1:2, and 1:3. The draw ratio (DR, final fiber length/initial fiber length) for each configuration is called DR1 (undrawn), DR2, and DR3. Five samples (*n* = 5) were collected for each draw ratio.

### 2.4 Yarn Spinning Process

The yarn spinning process is shown in Figure 1D,E. Rolls of aligned and overlapping nanofibers are fed into a yarn spinning device consisting of a twisting motor and a winding motor attached to a bobbin. The twist per inch (TPI) is the ratio of the twisting speed to the winding speed and can be controlled by changing the speed of the motors. A resulting yarn is shown in Figure 1F. A video showing the full production process can be viewed in the Supporting Information.

### 2.5 Mechanical Testing

The mechanical properties of the nanofiber yarns were investigated using a single-strand method. Testing was conducted on a Shimadzu EZ-SX universal testing machine using a 100 N load cell and 1 kN capstan yarn grips. Testing was performed using a modified version of the ASTM D2256 standard. Strain rate was set to 10 mm/min, and yarns were loaded to have a gauge length of 10 mm. All tests were conducted until yarn failure. Control fibers (not spun into yarns) were also tensile-tested. Collected fibers were adhered to plastic frames and loaded onto mechanical grips. Testing was carried out using a 100 N load cell at a strain rate of 4.5 mm/min until fiber failure.

The total cross-sectional area of each fiber control sample was calculated by multiplying the total number of fibers by the average individual fiber cross-sectional area, which was measured using scanning electron microscopy (SEM). Yield stress was identified as the point where the stress–strain curve transitioned from the linear to the nonlinear or plastic region.^20^ Frame-mounted nanofiber arrays evaluate the effect of postdrawing on individual fiber mechanics and allow comparison of component fiber mechanics to yarn mechanics.

The linear density of yarns was calculated by measuring the mass of yarn segments after 48 h of drying and dividing the mass by the length. Specific strength was calculated by dividing the maximum force by the linear density. The stress–strain curves of yarns were estimated by using the linear density of each yarn and the bulk density of PVDF-HFP to estimate the total cross-section.

### 2.6 Fourier Transform Infrared (FTIR) Spectroscopy

The chemical composition of PVDF-HFP nanofiber yarns was quantitatively evaluated by using FTIR spectroscopy. A Thermo Nicolet Nexus 670 FTIR spectrometer was employed for this purpose. Attenuated total reflectance (ATR) mode was used to evaluate yarn samples, and transmission mode was used to evaluate mounted nanofiber arrays. The OMNIC Software tool established the baseline for wavelength and absorbance at an observed spectra region between 400 and 4000 cm^–1^. All spectra were normalized to the C–C stretching peak at 1070 cm^–1^.

Equation 1, known as the Lambert–Beer law equation, can be used to calculate the associated piezoelectric β-phase content. Here, *X*_α_ is the mass fraction of the α phase and *X*_β_ is the crystal mass fraction of the β phase. *A*_α_ and *A*_β_ are the absorbance band intensities. *K*_α_ and *K*_β_ are the absorption coefficients.^20,28^ β-phase content was calculated using Beer–Lambert’s law with the β-phase peak at 840 cm^–1^ and the α-phase peak at 761 cm^–1^ to provide key insights into the piezoelectric properties of nanofibers and nanoyarns.
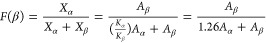
1

### 2.7 SEM

A scanning electron microscope (FEI Apreo 2) was used to image yarn and fiber samples in order to measure their diameter. Nanofibers were collected on 10 × 10 mm plastic window frames and carefully mounted on carbon-taped aluminum stubs. After sputter-coating, samples were imaged at 2000×, 6500×, and 10,000× magnifications. ImageJ was used to estimate the number of fibers mounted on frames and to precisely measure the fiber and yarn diameters.

### 2.8 Mechanical-Electrical Experimental System

PVDF-HFP yarns were tested for piezoelectric response using two deformation methods: bending (flick) and axial (stretch) methods. For the bending method, a rotating wheel, with six evenly separated flexible plastic tabs extending out, strikes the yarn as it rotates. For axial stretching, a 4 cm yarn sample is attached to a rotating motor shaft on one end, with the other end fixed. Motor movement produces cyclic stretching as the shaft rotates clockwise and then counterclockwise at 93 rpm, winding and then unwinding the yarn. Both setups used conductive paint and wires as leads for voltage signal detection across a 10 mm segment of the PVDF-HFP yarn. A data acquisition system (DAQ, National Instruments USB-6001) with LabView software was used to capture the peak-to-peak voltage across the leads, and the average was used as a quantitative measure of piezoelectric performance. The output signal was not amplified. The tests were run for 1 min on yarns with different nanofiber materials, DRs, ages, and treatments.

### 2.9 Statistical Analysis

Group-level analyses were performed across all measured parameters, calculating averages and standard deviations, using Microsoft Excel. Python libraries (NumPy, Pandas, and Matplotlib) were employed for data processing and visualization. Statistical validity was ensured through ANOVA tests and *t* tests performed for group-to-group analysis using the SciPy library.

## 3 Results and Discussion

### 3.1 Morphology Analysis

**Figure 2:**
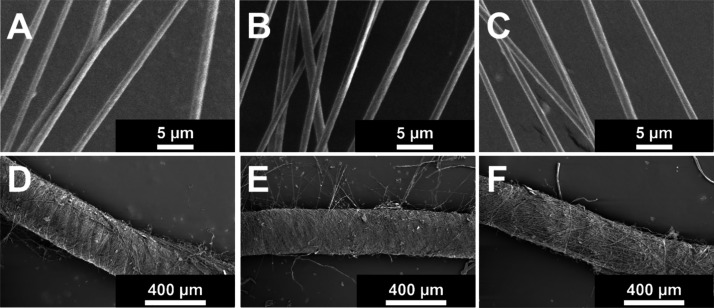
(A–C) Representative SEM images of PVDF-HFP nanofibers with fiber DRs of DR1–DR3, respectively. (D–F) Representative SEM images of PVDF-HFP nanoyarns spun from nanofibers with DRs of DR1–DR3, respectively.

The SEM images of nanofibers and yarns (DR1, DR2, and DR3) collected using an automated track device show that the fibers have an aligned orientation. Nanofiber and nanoyarn diameters across different samples and processing conditions are summarized in Table 1. Representative SEM images are shown in Figure 2. Fiber diameters were similar for all DRs from DR1 (1.12 ± 0.15 μm) to DR2 (1.08 ± 0.11 μm) and DR3 (1.07 ± 0.12 μm). It is expected that fiber diameter will decrease with an increasing DR. However, it is also expected that fiber diameter will decrease with increasing top gap size.^29^ We hypothesize that the competing effects of the larger top gap for smaller DRs (DR1 = 12 cm, DR2 = 6 cm, DR3= 4 cm) resulted in a similar final diameter for all three DRs. Yarn diameters decreased slightly with increasing DR of the component fibers, ranging from 429 ± 11.2 μm (DR1) to 352 ± 27.5 μm (DR3).

**Table 1:** Summary of Fiber and Yarn Diameters and Yarn Linear Densities

**sample type**	**average diameter (μm)**	**linear density (den)**
DR1 (fiber)	1.12 ± 0.15	N/A
DR2 (fiber)	1.08 ± 0.11	N/A
DR3 (fiber)	1.07 ± 0.12	N/A
DR1 (yarn)	429 ± 11.2	817 ± 145
DR2 (yarn)	374 ± 21.3	1130 ± 306
DR3 (yarn)	352 ± 27.5	1200 ± 173

### 3.2 Mechanical Properties

#### 3.2.1 PVDF-HFP Nanofiber Mechanics

**Figure 3:**
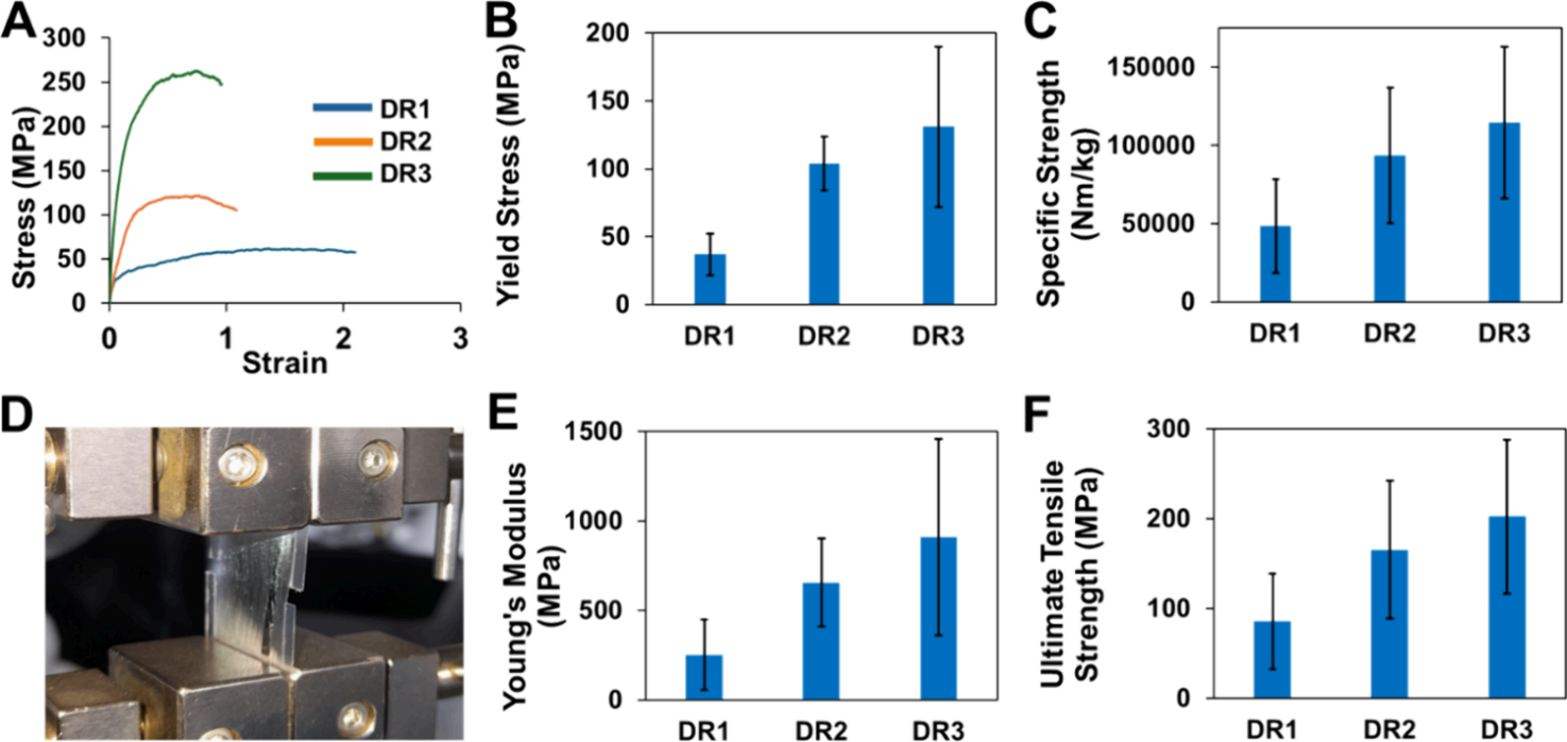
Mechanical behavior of component nanofibers vs DR (DR1, DR2, and DR3). (A) Representative stress–strain curves. (B) Yield stress. ANOVA *p*-value < 0.01, and all group-to-group comparisons with Student's *t* tests give a *p*-value of <0.01, except for groups DR2 vs DR3. (C) Specific strength. ANOVA *p*-value = 0.01, and group-to-group comparisons with Student's *t* test have a *p*-value of <0.01, except for groups DR2 vs DR3. (D) Image of the tensile test of an aligned nanofiber array with the sides of the frame cut prior to testing. (E) Young’s modulus. ANOVA *p*-value > 0.01, and all group-to-group comparisons with Student's *t* tests give a *p*-value of >0.01 (F) Ultimate tensile strength. ANOVA *p*-value of >0.01, and all group-to-group comparisons with Student's *t* tests give a *p*-value of >0.01. All averages and standard deviations (B,C,E,F) are based on five replicates (*n* = 5).

The mechanical behavior of component PVDF-HFP nanofibers, which make up the nanoyarns, shows a trend of increasing yield stress, specific strength, Young’s modulus, and ultimate tensile strength as the DR increases from DR1 to DR3 (Figure 3). Ultimate tensile strength increases from 85.9 MPa for DR1 to 165 MPa for DR2 (92% increase) and 203 MPa for DR3 (23% increase from DR2). Young’s modulus increases from 253 MPa for DR1 to 656 MPa for DR2 (159% increase) and 911 MPa for DR3 (39% increase from DR2). Yield stress increases from 36.9 MPa for DR1 to 104 MPa for DR2 (182% increase) and 131 MPa for DR3 (26% increase from DR2). Nanofiber specific strength increases with increasing DR, resulting in DR1 at 48,538 N m/kg, DR2 at 93,455 N m/kg (a 93% increase compared to DR1), and DR3 at 114,446 N m/kg (a 22% increase compared to DR2 and 136% compared to DR1). The increase in specific strength of the fibers is attributed to postdrawing effects such as enhanced molecular alignment and/or crystallinity.

#### 3.2.2 PVDF-HFP Nanoyarn Mechanics

**Figure 4:**
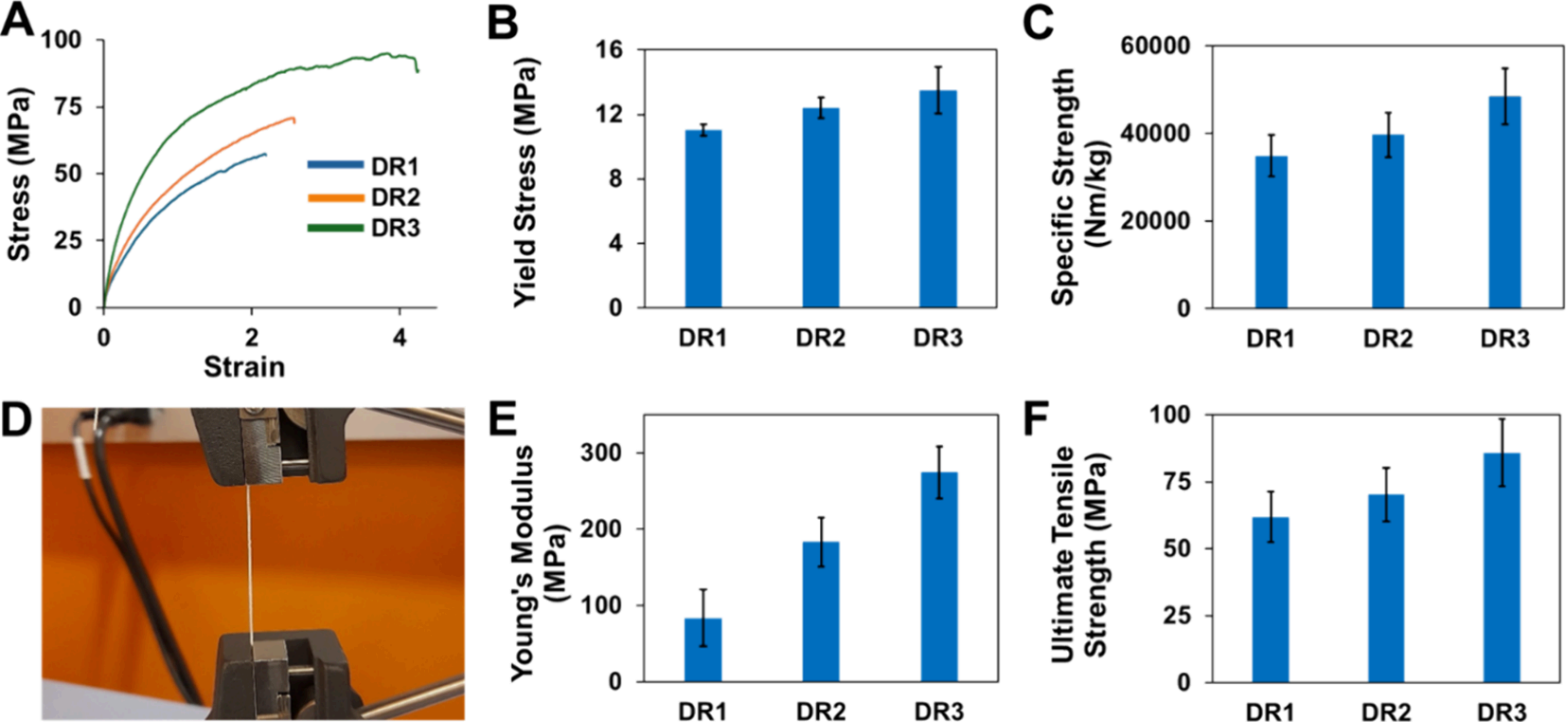
Mechanical behavior of nanoyarns vs the DR of the component nanofibers making up the yarn (DR1, DR2, and DR3). (A) Representative stress–strain curves. The curves are truncated after the ultimate tensile strength peak for a better presentation. (B) Average yield stress. ANOVA *p*-value of <0.01, and all group-to-group comparisons with Student's *t* tests give a *p*-value of <0.01, except for groups DR2 vs DR3. (C) Average specific strength. ANOVA *p*-value of <0.01, and group-to-group comparisons with Student's *t* test give a *p*-value of <0.01 for DR1 vs DR3 only. (D) Image of a tensile test of a nanofiber yarn. (E) Young’s modulus. ANOVA *p*-value of <0.01, and all group-to-group comparisons with Student's *t* test give a *p*-value of <0.01, except for groups DR1 vs DR3. (F) Ultimate tensile strength. ANOVA *p*-value of <0.01, and group-to-group comparisons with Student's *t* tests have a *p*-value of <0.01 for DR1 vs DR3 only. All averages and standard deviations (B,C,E,F) are based on five replicates (*n* = 5).

The effect of the component fiber DR on nanoyarn mechanics is shown in Figure 4. Nanoyarns exhibit improved mechanical properties with increasing DRs. Specifically, DR1 yarns had an ultimate tensile stress of 61.8 MPa, while DR2 yarns showed a 13.6% increase to 70.2 MPa, and DR3 yarns demonstrated a further 22.3% increase to 85.9 MPa. DR1 yarns had the lowest Young’s modulus at 83.4 MPa, while DR2 yarns showed an 84.2% increase to 154 MPa, and DR3 showed a further 45.2% increase to 224 MPa. For yield stress, DR1 had 11.0 MPa, DR2 increased to 12.4 MPa (a 12.7% rise), and DR3 reached 13.5 MPa (an 8.8% increase). The specific strength of the PVDF-HFP nanoyarn also increased with higher DRs. DR1 yarns had a specific strength of 34,900 N m/kg compared to 39,700 N m/kg for DR2 (a 13.8% increase) and 48,500 N m/kg for DR3 (22.1% increase from DR2 and 39.0% from DR1). The increase in yarn strength with increasing DR is much less pronounced than the increase in strength of individual nanofibers shown in Figure 3C. This is likely due to the failure mode of the nanoyarn, which may be mediated by unraveling rather than fiber failure. Sources investigating the mechanics of the PVDF-HFP yarns are scarce. However, the ultimate tensile values for PVDF-HFP twisted yarns have been reported between 4 and 15 MPa,^17^ which is four times lower those measured in this study.

### 3.3 FTIR Analysis

**Figure 5:**
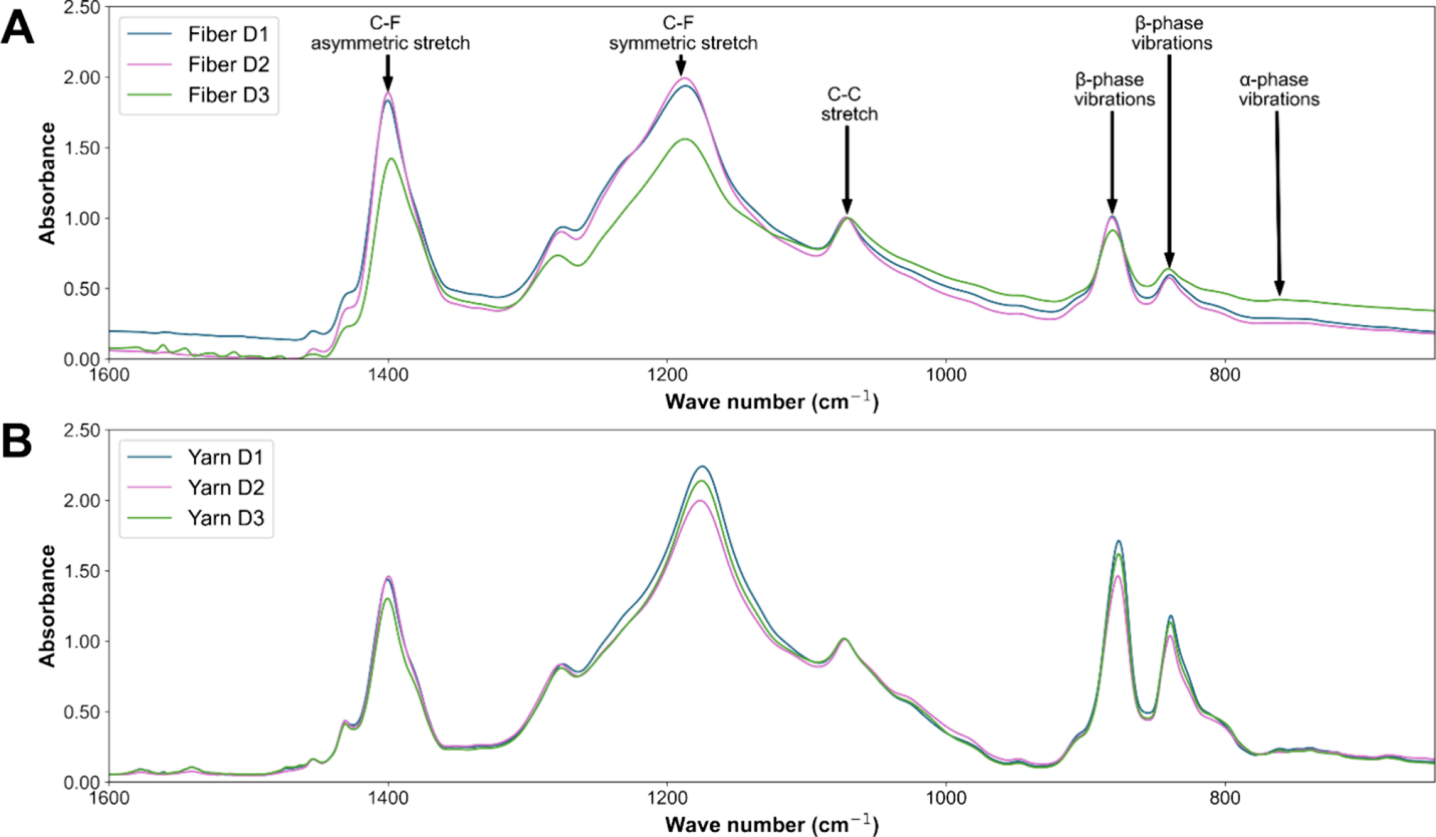
(A) Representative FTIR spectra for PVDF-HFP nanofibers post drawn at DR1, DR2, and DR3. (B) Representative FTIR spectra for PVDF-HPF nanoyarns spun from DR1, DR2, and DR3 nanofibers.

The FTIR spectra for all yarn and fiber groups are shown in Figure 5. The β-phase content was calculated using eq 1 and using absorption values for the β-phase peak at 840 cm^–1^ and the α-phase peak at 761 cm^–1^. Average β-phase contents are summarized in Tables 2 and 3.

**Table 2:** Average β-Phase Contents of Nanofibers (*n* = 5)^a^

average β-phase content (nanofiber)			
film	DR1	DR2	DR3
0.417–0.660^20,30,31^	0.942	0.939	0.857

a ANOVA *p*-value of <0.01, and group-to-group comparisons with Student's *t* test give a *p*-value of <0.01 for DR1 vs DR3 and DR2 vs DR3.

**Table 3:** Average β-Phase Contents of Nanoyarns (*n* = 5)^a^

average β-phase content (nanoyarn)			
film	DR1	DR2	DR3
0.417–0.660^20,30,31^	0.966	0.973	0.971

a ANOVA *p*-value of <0.01. When nanofiber and nanoyarns with matched DR are compared, Student's *t* test gives a *p*-value of <0.01 for DR1, DR2, and DR3.

The β-phase content, a key indicator of piezoelectric efficiency, was substantially higher for all electrospun nanofibers compared to the values reported in the literature for PVDF-HFP films.^20,30,31^ Nanofiber β-phase content increased slightly as the DR increased from DR1 to DR2 and then decreased from DR2 to DR3 (0.924, 0.939, and 0.857, respectively).

Nanoyarns had even higher β-phase content than the component nanofibers (*p* < 0.01 for all DRs). In contrast to nanofibers, there was no notable change in β-phase content as the component fiber DR increased from DR1 to DR2 to DR3 (0.966, 0.973, 0.971, respectively). The elevated β-phase content in yarns compared to their prespun component fibers could be due to the mechanical forces and stretching that occurs during the yarn spinning process.

### 3.4 Piezoelectrical Testing Analysis

#### 3.4.1 Cyclic Deformation Testing

**Figure 6:**
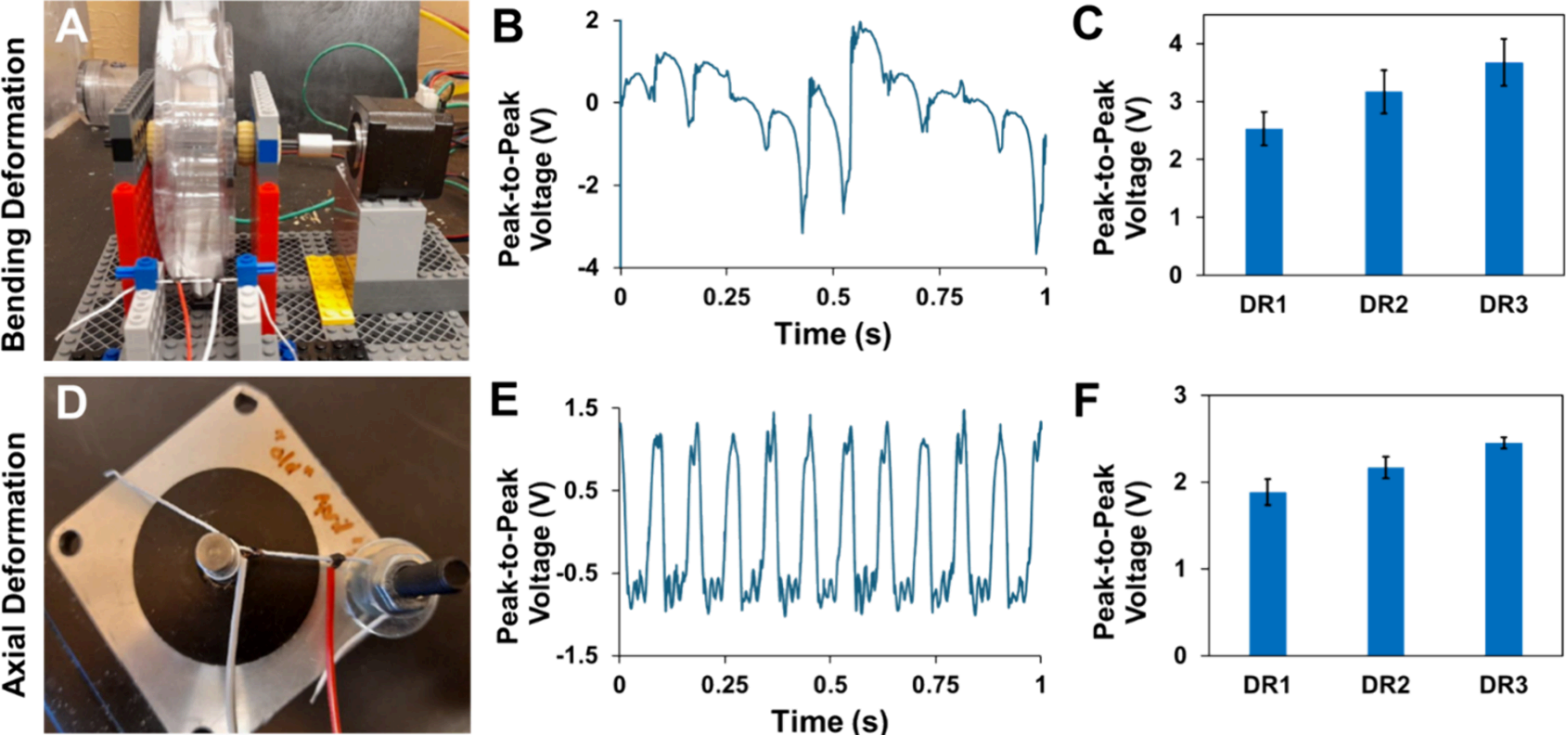
(A) Bending deformation (flick) test on an electrospun PVDF-HFP nanoyarn sample using a rotating wheel with six tabs sticking out along the circumference to contact the suspended yarn segment. (B) A representative graph of peak-to-peak voltage outputs over time for the bending deformation test. (C) Average peak-to-peak voltage per DR. ANOVA *p*-value <0.01, and group-to-group comparisons with Student's *t* test give a *p*-value of <0.01 for group DR1 vs DR3 only. (D) Axial deformation (stretch) test on an electrospun PVDF-HFP nanoyarn sample using a programmable rotating motor shaft to induce cyclic axial displacement. (E) A representative graph of peak-to-peak voltage outputs over time for the stretch deformation test. (F) Average peak-to-peak voltage per DR. ANOVA *p*-value of <0.01, and all group-to-group comparisons with Student's *t* test give a *p*-value of <0.01. All averages and standard deviations (C,F) are based on five replicates (*n* = 5).

The bending deformation (flick) test results, as illustrated in Figure 6A–C, are uneven but demonstrate a clear correlation between the component nanofiber DR and the average peak-to-peak voltage generation. We expect that the unevenness of the signal is the result of uneven deformation associated with the experimental setup. The average peak-to-peak voltage exhibits an increasing trend with higher DRs: 2.53 V for DR1, 3.17 V for DR2, and 3.67 V for DR3. This pattern suggests that elevated component fiber DRs contribute to enhanced voltage generation in the yarns. The results from axial deformation tests, shown in Figure 6D–F, are much more consistent and reveal a positive correlation between the component nanofiber fiber DR versus yarn voltage generation. The average peak-to-peak voltage increases with higher DRs: 1.88 V for DR1, 2.17 V for DR2, and 2.44 V for DR3.

The piezoelectrical output of a PVDF-HFP yarn could be affected by several factors, such as changes in macromolecular composition and organization (such as crystal fraction and chain alignment), fiber alignment, cross-sectional area, and surface area to volume ratio. The first factor to look at is the β-phase crystal fraction; however, this is substantially similar for all three groups. Fiber diameters are also similar for all three groups. Two possible explanations could be (1) the higher packing density (ratio of linear density to yarn diameter, Table 1) of DR2 and DR3 yarns or (2) enhanced macromolecular alignment associated with postdrawing. The electrical output of PVDF-HFP in the post drawn groups falls within the ranges observed in other studies of electrospun PVDF-HFP nanofiber devices.^32−35^ The electrical output of the devices depends on both the piezoelectric material and the overall device design. Notably, increasing DRs led to systematic improvements in energy conversion despite no apparent increase in β-phase content. This enhanced performance may stem from better crystalline alignment made possible by postdrawing, resulting in increased overall piezoelectricity of the samples^20^ or higher yarn packing density, which improves the transfer of charge to the testing leads.

#### 3.4.2 Long-Term Stability

To determine the long-term stability of PVDF-HFP yarns, samples previously tested in Section 3.3 were retested after approximately one year (Table 4). The peak-to-peak voltage decreased to 0.61 V, which was similar to a freshly electrospun nonpiezoelectric polycaprolactone (PCL) yarn, used as a control. New PVDF-HFP yarns were fabricated to investigate this further. They were tested at 24 h and again at 1 week. This new batch of yarns (Batch #2) had a similar response, with higher peak-to-peak voltage (19.1 V) that decreased only slightly after 1 week (18.8 V). However, immersing these samples in deionized (DI) water, to remove surface charge, resulted in a diminished peak-to-peak voltage similar to nonpiezoelectric PCL and a-year-old PVDF-HFP yarns.

**Table 4:** Peak-to-Peak Voltages for Yarns under Axial Deformation

	**PVDF-HFP yarn (Batch #2)**			**PCL yarn**		**PVDF-HFP yarn (Section 3.4)**	
parameter	24 h	1 week	DI quench	24 h	DI quench	3–5 weeks	1 year
peak-to-peak voltage (V)	19.1	18.8	0.75	0.73	0.6	2.3	0.61

Based on these results, we hypothesize that the residual surface charge from electrospinning allows piezoelectric dipoles to generate current along the length of the yarn that are measurable over a gap (10 mm between leads). Nonpiezoelectrical materials are not able to generate current even with the presence of residual surface charge. We hypothesize that piezoelectric dipoles persist in 1-year-old yarns; however, they cannot be detected without surface charge to facilitate current flow to the spaced electrodes used in our instrumentation setup. The smaller peak-to-peak voltage (0.6–0.75 V) generated by nonpiezoelectric yarns and piezoelectric yarns with surface charge removed by DI water is likely the result of tribological effects. These findings underscore the crucial role of charge transport in enabling the effective use of piezoelectric yarns in sensing and energy harvesting devices. Integrating conductive materials into piezoelectric yarns may facilitate current generation along their length even in the absence of surface charge. Additionally, placing leads in close proximity could enable sensing and energy harvesting without relying on the surface charge.

## 4 Conclusions

This research presents a significant advancement in the production of electrospun \PVDF-HFP nanofiber yarns using a novel continuous manufacturing approach. This study demonstrates the effectiveness of an automated parallel track system and an adjustable roll-to-roll collector in producing PVDF-HFP nanoyarns with enhanced mechanical and piezoelectric properties. The postdrawing process, applied to individual fibers before yarn spinning, proved crucial to improving tensile strength and piezoelectric outputs of the nanofiber yarns. This study found evidence that the residual charge on nanofibers after electrospinning was essential to promote current flow to testing leads separated by a 10 mm distance. This is an important consideration for the PVDF-HFP device design.

The results highlight the potential for scaling up the production of high-performance electrospun PVDF-HFP nanofiber yarns for commercial applications. Integrating postdrawing with electrospinning, roll-to-roll collection, and yarn spinning in a stepwise, but continuous, manufacturing process addresses limitations of previous self-bundling methods, such as individual fiber processing and continuous production constraints. The study’s findings have substantial implications for the development of smart textiles and wearable devices, as the enhanced piezoelectric properties of these nanofiber yarns make them ideal for integration into intelligent fabrics and flexible electronics.

## Supporting Information Available

A video showing the full production process (MP4)
